# Spatiotemporal use predicts social partitioning of bottlenose dolphins with strong home range overlap

**DOI:** 10.1002/ece3.4681

**Published:** 2018-12-11

**Authors:** Rodrigo C. Genoves, Pedro F. Fruet, Juliana C. Di Tullio, Luciana M. Möller, Eduardo R. Secchi

**Affiliations:** ^1^ Museu Oceanográfico ‘Prof. Eliézer de C. Rios’ Rio Grande RS Brazil; ^2^ Laboratório de Ecologia e Conservação da Megafauna Marinha – EcoMega, Instituto de Oceanografia Universidade Federal do Rio Grande (FURG) Rio Grande RS Brazil; ^3^ Programa de Pós‐Graduação em Oceanografia Biológica Universidade Federal do Rio Grande (FURG) Rio Grande RS Brazil; ^4^ Kaosa Rio Grande RS Brazil; ^5^ Cetacean Ecology, Behaviour and Evolution Laboratory Flinders University Adelaide SA Australia; ^6^ Molecular Ecology Laboratory Flinders University Adelaide SA Australia; ^7^ Centro Nacional de Pesquisa e Conservação de Mamíferos Aquáticos – CMA ICMBio/MMA Santos SP Brazil

**Keywords:** affiliation, gregariousness, social division, social group, spatial distribution, spatiotemporal dynamics

## Abstract

Ranging behaviour and temporal patterns of individuals are known to be fundamental sources of variation in social networks. Spatiotemporal dynamics can both provide and inhibit opportunities for individuals to associate, and should therefore be considered in social analysis. This study investigated the social structure of a Lahille's bottlenose dolphin (*Tursiops truncatus gephyreus*) population, which shows different spatiotemporal patterns of use and gregariousness between individuals. For this, we constructed an initial social network using association indices corrected for gregariousness and then uncovered affiliations from this social network using generalized affiliation indices. The association‐based social network strongly supported that this dolphin population consists of four social units highly correlated to spatiotemporal use patterns. Excluding the effects of gregariousness and spatiotemporal patterns, the affiliation‐based social network suggested an additional two social units. Although the affiliation‐based social units shared a large part of their core areas, space and/or time use by individuals of the different units were generally distinct. Four of the units were strongly associated with both estuarine and shallow coastal areas, while the other two units were restricted to shallow coastal waters to the south (SC) and north of the estuary (NC), respectively. Interactions between individuals of different social units also occurred, but dolphins from the NC were relatively more isolated and mainly connected to SC dolphins. From a conservation management perspective, it is recommended that information about the dolphin social units should be incorporated in modeling intrapopulation dynamics and viability, as well as for investigating patterns of gene flow among them.

## 1 INTRODUCTION

Social structure is a synthesis of the nature, quality, and patterning of the relationships among members of a population (Hinde, 1976). Therefore, the way that a population is structured is a key component of its biology, genetics and spatiotemporal dynamics, representing an important factor in management and conservation of wildlife (Whitehead, 2008a). Regarding social organization, individuals can associate with either the same or with several different individuals over time. In mammals, stable groups are usually observed in matrilineal societies (Whitehead, 2003), whereas in fission–fusion societies wide variation in group size and/or composition is usually observed, along with temporal variation in spatial cohesion (Aureli et al., 2008). Fission–fusion social dynamics are commonly found in some societies of primates (van Schaik, 1999), dolphins (Connor, Wells, Mann, & Read, 2000), bats (Kerth, Ebert, & Schmidtke, 2006), and elephants (Wittemyer, Douglas‐Hamilton, & Getz, 2005).

Although there is much fluidity in the individual associations within populations governed by fission–fusion dynamics, on a fine‐scale these populations can be structured into social units (Best, Seddon, Dwyer, & Goldizen, 2013; Karczmarski, Würsig, Gailey, Larson, & Vanderlip, 2005; Urian, Hofmann, Wells, & Read, 2009). Social segregation of individuals may be related to common biological and behavioral factors such as sex, age, feeding strategy, behavior, habitat use, or preferential/avoided companions (Krause & Ruxton, 2002). Therefore, social units usually arise when some individuals of a population are largely behaviorally self‐contained, interacting more with each other than with others, sharing a similar living space, and generally use this space at the same time (Whitehead, 2008a). These imply that in a population with social units, individuals can present different spatiotemporal use patterns. The challenge when describing this kind of social system is thus to define an appropriate spatiotemporal scale within which the social patterns can be adequately described (Cantor et al, 2012).

Most studies about social networks of nonhuman populations have been based on matrices of association indices, which estimates the proportion of time pairs of individuals stay associated, and these are used to define social units (Whitehead, 2008a). However, to access preferred and avoided dyadic relationships from association data (also called true affiliations), and the structural factors that may affect associations, have been a major challenge for behavioral ecologists (Bejder, Fletcher, & Bräger, 1998; Croft, Madden, Franks, & James, 2011; Godde, Humbert, Côté, Réale, & Whitehead, 2013; Whitehead & James, 2015). These factors can be related, for example, to spatial overlap (Shizuka et al., 2014), temporal overlap (Cantor et al., 2012), gregariousness (Godde et al., 2013), and sex of individuals (Wiszniewski, Lusseau, & Möller, 2010). To deal with multiple structural factors affecting association indices, Whitehead and James (2015) proposed the use of residuals following a multiple regression on the association indices and on structural variables using generalized linear models, which they called generalized affiliation indices (GAIs). Both GAIs and association indices can be used for network analysis to understand the social structure of animals, either at an individual or population level (Croft, James, & Krause, 2008; Farine & Whitehead, 2015).

Bottlenose dolphins, *Tursiops* spp., are cosmopolitan animals that inhabit coastal and oceanic waters of both tropical and temperate regions (Wells & Scott, 1999). Studies around the world, mainly on coastal animals, have demonstrated that fission–fusion social dynamics appear to be the rule for bottlenose dolphins (Connor et al., 2000), although some populations contain stable components (Lusseau et al., 2003; Wells, 2014). Factors that can be associated to the structuring of social units within bottlenose dolphin populations include the association patterns of individuals (Lusseau et al., 2006; Wiszniewski, Allen, & Möller, 2009), ranging patterns (Rossbach & Herzing, 1999; Urian et al., 2009), feeding strategies (Ansmann, Parra, Chilvers, & Lanyon, 2012; Chilvers & Corkeron, 2001; Daura‐Jorge, Cantor, Ingram, Lusseau, & Simões‐Lopes, 2012; Mann, Stanton, Patterson, Bienenstock, & Singh, 2012), habitat use (Baird et al., 2009; Laska, Speakman, & Fair, 2008), sex (Wiszniewski, Brown, & Möller, 2012), and kinship relationships (Möller, Beheregaray, Allen, & Harcourt, 2006; Möller, Castaing, Salomon, & Lazure, 2001; Parsons et al., 2003).

Bottlenose dolphins from subtropical coastal waters of the western South Atlantic hold unique morphological and genetic characteristics compared to their offshore counterparts (Costa, Rosel, Daura‐Jorge, & Simões‐Lopes, 2016; Fruet et al., 2017; Wickert, Eye, Oliveira, & Moreno, 2016). These dolphins were recently recognized as a new dolphin subspecies, the Lahille's bottlenose dolphin, *Tursiops truncatus gephyreus* (Committee on Taxonomy, 2017) (although these characteristics have been argued to be indicative of species‐level differences by some authors; Wickert et al., 2016). Some populations of the Lahille's bottlenose dolphins have also been proposed as discrete management units, such as in the Patos Lagoon Estuary (PLE) and adjacent coastal waters (Fruet et al., 2014, 2017 ). Recent mark‐recapture studies using photo‐identification (photo‐ID) to individually recognize dolphins through natural marks on their dorsal fins have demonstrated that a small, relatively stable, resident population of ~87 individuals inhabit the sheltered waters of the PLE in southern Brazil (Fruet, Daura‐Jorge, Möller, Genoves, & Secchi, 2015a; Fruet, Secchi, Tullio, & Kinas, 2011). It is noteworthy that these studies were restricted to resident individuals using PLE and did not include individuals sighted using adjacent coastal waters. Although this portion of the population has remained stable, the population as a whole has over the years suffered unnatural mortality associated with fishing activities (Fruet et al., 2012), and changed its feeding ecology (Secchi et al., 2016) due to overfishing and habitat degradation (Moraes, Paes, Garcia, Möller, & Vieira, 2012). Studies on spatial use patterns of this population, considering both the PLE and adjacent coastal waters, showed a preference of individuals for waters around the estuary mouth and its vicinities, as well as adjacent shallow (depth ≤ 6 m) coastal waters (Di Tullio, Fruet, & Secchi, 2015; Mattos, Dalla Rosa, & Fruet, 2007). Di Tullio et al. (2015) also found a decrease in dolphin densities in the southern coastal area during warmer months, possibly associated with increased anthropogenic disturbance during this period. However, these studies show spatiotemporal use patterns at the population level, which is unlikely to be enough for effective conservation management of socially structured populations. On an individual scale, preliminary analyses revealed that some individuals appear to not enter estuarine waters. Among dolphins that were never observed inside the estuary, some appear to travel during the colder months from Uruguay to PLE's adjacent southern coast (*ca* 250 km southward; Laporta et al., 2016), while others, tend to use the area immediately to the north of the PLE during warmer months (R.C.G., personal observation).

The objectives of this long‐term study on this Lahille's bottlenose dolphin population were to (a) categorize and group individuals according to their patterns of spatial use and temporal fidelity to the area; (b) identify the most adequate analytical method to describe its social structure; and (c) verify the presence of social units and elucidate their role within the population's social network.

## 2 METHODS

### 2.1 Study area and data collection

**Figure 1:**
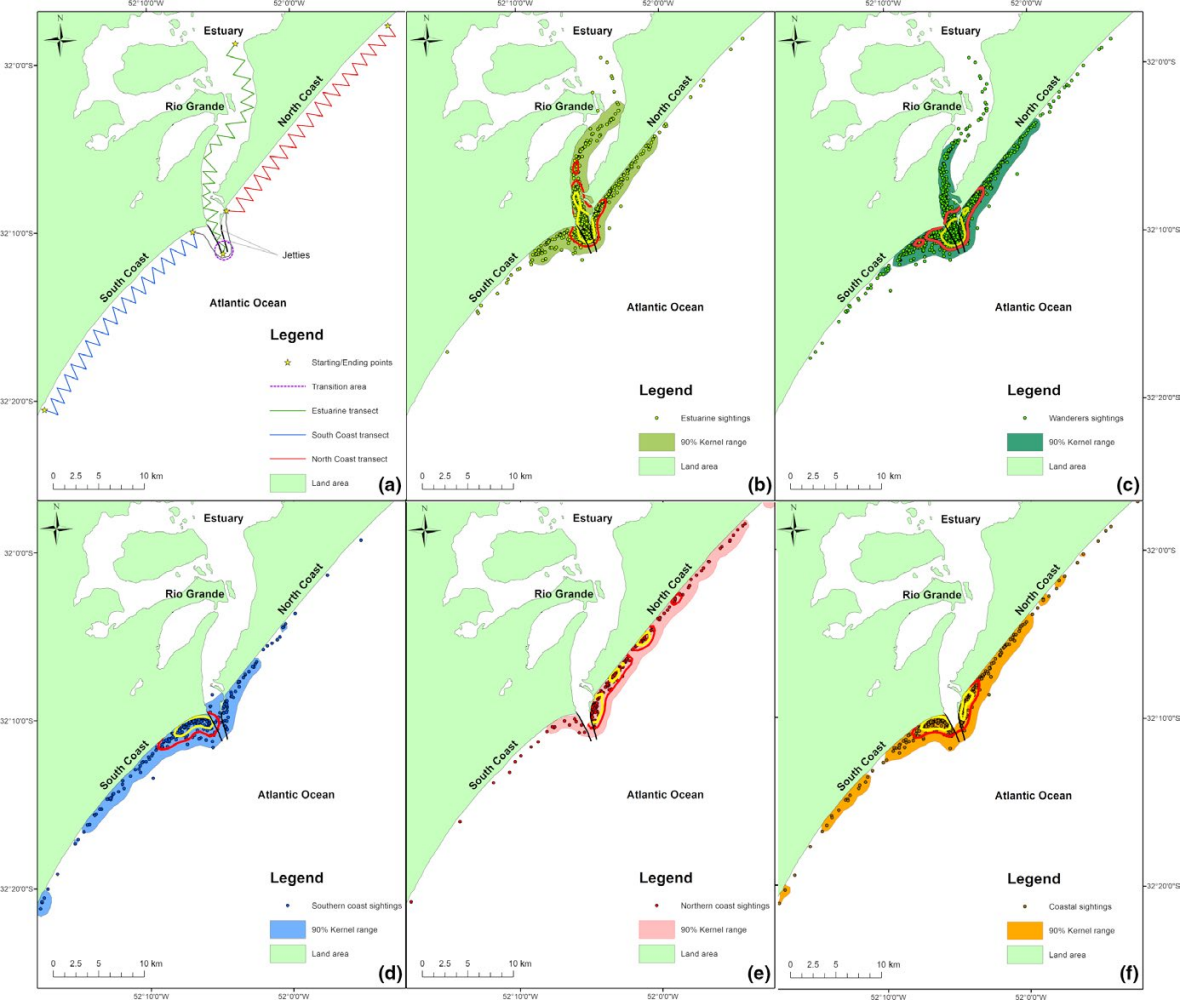
(a) Area covered during boat surveys (sampling occasions) to search for Lahille's bottlenose dolphins (*Tursiops truncatus gephyreus*) in the Patos Lagoon Estuary (green) and adjacent coastal waters (jetties transect = gray, south = blue and north = red) in southern Brazil. The dotted purple circle in the mouth of the estuary represents the transition area. (b–f) Locations where dolphins (grouped by their spatial preferences) were photographed within the study area are plotted separately, with the 90% (full color), 50% (red line) and 25% (yellow line) kernel isopleths for each group (estuary (b), wanderers (c), south coast (d), north coast (e), and coastal (f) dolphins)

The Patos Lagoon is a large coastal lagoon located between 30°30′S and 32°12′S (*ca* 10,000 km^2^). It is a subtropical system that receives freshwater input from a drainage basin of about 200,000 km^2^ in southern Brazil (Möller et al., 2001), and is connected to the Atlantic Ocean by two jetties of about 4 km. Approximately 10% of the area is characterized as an estuary composed of shallow bays (80% of which are <2 m in depth), and a narrow navigation channel that can reach up to 20 m deep. The Patos Lagoon Estuary (PLE) is one of the most productive fishing grounds in Brazil, with abundant assemblages of fish in the estuary and adjacent coastal waters (Garcia, Vieira, Winemiller, Moraes, & Paes, 2012; Rodrigues & Vieira, 2013). Our study area includes the lower part of the PLE and adjacent coastal waters (*ca* 140 km^2^) (Figure 1a). The area immediately south of the estuary mouth consists of a dissipative beach, with mainly mud and sandy mud originated from the estuarine plume. The beach to the north is characterized as more reflective and with larger particle sizes compared to the south (Figueiredo & Calliari, 2006). For the purpose of survey design and due to some logistical limitations, the area was divided into three subareas: (a) the estuary to the lagoon's mouth (*ca* 40 km^2^); (b) the estuary's adjacent northern coastal waters; and iii) the estuary's adjacent southern coastal waters. The two coastal areas are ~50 km^2^ each and are strongly influenced by the surf zone (Figure 1a). Furthermore, due to the characteristics of the area, with a triple intersection of subareas, a transition area was created, mainly to prevent individuals transiting between the coastal areas in front of the estuary mouth to be designated as "sighted in the Estuary". This transition area was defined as a circumference of 1,000 m radius, centered on the median of an imaginary line between the end of the two jetties of the PLE (Figure 1a).

The surveys were conducted between January 2006 and December 2015 onboard a 5 m boat powered with a 90 hp outboard engine, with at least three people on board: a skipper, a photographer, and a note taker. All three were responsible for estimating the minimum (the lower value among them), maximum (highest value among them), and best group size (through a consensus decision). Surveys were restricted to favorable weather conditions (i.e., Beaufort ≤3, good visibility, and swell <2 m). Zig‐zag transects were run through the estuary in all sampling occasions (Figure 1a). The coastal areas were initially surveyed through transects perpendicular to the coastline, in order to investigate the width of the population's spatial use patterns on the coast. During these surveys, it was observed that bottlenose dolphins were only rarely found beyond two nautical miles from the shore (Di Tullio et al., 2015). Therefore, after the identification of this core coastal area in February 2012, the southern and northern coastal areas were surveyed with zig‐zag transects from the coastline to 1.5 nm offshore for the remainder of the study (Figure 1a). Each survey covered at least one of the three subareas. At least one survey per month was conducted in each subarea, and each of them had two different starting points, closest or farthest from the estuary's mouth (see Figure 1a). These were alternated to diversify the route and reduce possible bias in the data collection due to sampling design.

Dolphins exhibiting spatial cohesion (i.e., within 100 m of each other) and that were engaged in similar activities were defined as a group (Wells, Scott, & Irvine, 1987). Time of sighting, group size, and geographic position (through a GPS) were recorded for each group sighted. In addition, individuals in a group were identified through evident long‐lasting marks (cuts and mutilations) and ancillary long‐lasting marks (nicks and deformities) in their dorsal fins using standard photo‐identification protocols (Urian et al., 2015). Other types of marks (e.g., tooth rakes, skin alterations) which are not long‐lasting were only used to assist in estimating the number of individuals in a group. Photographs were taken using a Nikon D300 digital camera equipped with a 300 mm lens. In subsequent analysis, each photograph was graded for quality (Q1–Q3; Wilson, Hammond, & Thompson, 1999). In excellent (Q1) photos, the dorsal fin was clearly visible (completely exposed), on sharp focus, oriented perpendicularly to the photographer and large enough to allow the detection of minor identifiable details. The use of lower quality photos (Q2 and Q3), where the fin is not fully visible, focus is somewhat blurry, and the angle not perpendicular, reduces the efficacy of the use of ancillary marks (e.g., minor cuts and deformities) and increases the probability of misidentification (false positive/negative; Friday, Smith, Stevick, & Allen, 2000). Since this was a systematic study, we chose, besides the use of evident long‐lasting marks, to use ancillary marks in the identification, increasing its reliability and allowing the use of individuals with only one evident long‐lasting mark (detailed further). For this reason, only Q1 photographs were considered in further analyses. Finally, two trained and experienced researchers independently identified all individuals “captured” (and “recaptured”) in these Q1 photographs, and then compared their results. In divergent events (two different IDs for one individual), both researchers repeated the process, comparing the photograph under analysis with the capture history (whole study period) of the two suggested individuals, until they reached a consensus. These primary data were recorded blindly because groups were photographed randomly, found within a predefined route, and the photo‐identification analysis was performed later by the two independent researchers.

### 2.2 Data treatment

The following analyses were restricted to dolphins with significant long‐lasting marks (i.e., at least two evident long‐lasting marks [cuts and/or mutilations], or one evident long‐lasting mark with at least two ancillary marks [nicks and/or deformities]) (allowing consistent matching between sampling periods), and that were photographed in at least ten sampling occasions, with at least five in the first half of the study (2006–2010) and five in the second half (2011–2015). Dolphins known to have died over the course of the study (i.e., found stranded on the beach) were excluded from analyses. These restrictions were adopted to ensure accurate identification, minimize the effects of sample size, to control for demographic effects and/or to control for the presence of rarely encountered individuals. Each survey which covered at least all transects of one of the areas (Figure 1a) was defined as a sampling occasion. Calves (e.g., <2 years old) were excluded from analyses as their association patterns cannot be considered independent from that of their mother. Groups where the number of individuals estimated in the laboratory, by photo‐id using only Q1 photographs, exceeded the maximum number of individuals estimated in the field (proving control of group size in the field), and groups in which the number of individuals estimated in the laboratory was less than half of the best‐estimated group size in the field (consensus decision among observers) were excluded from analysis (Lusseau et al., 2006).

#### 2.2.1 Data classification

##### Sex classification

The sex of individuals was obtained using (a) genetic sex determination from biopsy samples (only adult animals were sampled using modified darts specifically designed for small cetaceans (F. Larsen, Ceta‐Dart) fired from a 120‐lb draw weight crossbow, which has caused minor physical and behavioral disturbance in this population (see Fruet et al., 2016), following the protocol developed by Gilson, Syvanen, Levine, and Banks (1998); and (b) large dolphins (i.e., >3 m) with a closely associated calf photographed on ≥3 independent sampling occasions were determined as females (Fruet, Genoves, Möller, Botta, & Secchi, 2015b); and (c) large dolphins with several long‐lasting marks and scars in the dorsal fin which were first identified as adults in the first year of the study (2006) and never seen in close association with calves were determined as males.

##### Area classification

Each individually identified dolphin was classified as preferring a particular area (estuary—E, southern coast—S, or northern coast—N) based on where it was predominantly found (i.e.,>50% of all sightings in an area and <30% in the other two), excluding the transition area. This restriction on the frequency of sightings in other areas is to prevent an individual from being classified as, for example, an individual who predominantly uses the estuary, when in fact it also uses the southern area at similar frequency (e.g., 51% and 49%, respectively). In the case of coastal dolphins that do not enter the estuary and use only two areas, it was necessary for them to have more than 70% of sightings in one area to be classified as S or N dolphin. If an individual did not match any of these criteria, it was classified as a wanderer dolphin (W) (i.e., use all areas but has no area preference), or a coastal dolphin (C) if the individual did not use the estuary and showed no particular preference to one of the two coastal areas.

##### Period classification

In order to identify transient individuals, the study period was divided into Cold period (May to October) and Warm period (November to April). Dolphins sighted more than 70% of sampling periods (same criterion of two times adopted in the spatial class) in one of these periods were classified as transients (cold or warm) and those dolphins without a period preference as residents.

In order to verify the relevance of these classes as candidates for predictive variables of the GAIs, a Mantel test was conducted using SOCPROG 2.8 (Whitehead, 2009) to test if association indices were significantly higher between dolphins of the same class than between dolphins of other classes (Schnell, Watt, & Douglas, 1985).

### 2.3 Social analysis

The associations between individuals were based on group membership, such that dolphins present in the same group were assumed to be associated. The half‐weight index (HWI; Cairns & Schwager, 1987) was used to measure the intensity of the relationship between pairs of individuals. This index estimates the proportion of time that a given pair remains associated, is symmetric and varies between zero and one. It also enables comparisons between populations, and minimizes possible bias in the sample (e.g., misidentifications); therefore, it has been largely used in cetacean research (e.g., Whitehead, 2008b). The index is defined as: HWI = *x*/(*x* + *yab* + 0.5(*ya* + *yb*)), where, *x* is the number of sampling occasions in which the individuals *a* and *b* were observed in the same group; *yab* is the number of sampling occasions that *a* and *b* were identified in different groups; *ya* and *yb*, respectively, are the number of sampling occasions in which only the individuals *a* and *b* were identified. Unfortunately, the HWI does not account for differences in sociality or gregariousness among individuals in the population. Gregariousness exists when some individuals are found in consistently larger, or smaller, groups than others (Whitehead, Bejder, & Ottensmeyer, 2005), and this should be corrected because it can strongly affect the HWI (Godde et al., 2013). Typically, the presence of gregariousness can be tested by the Bejder et al. (1998) modification of the Manly (1995) procedure, which takes into account the standard deviation of the typical group size, which is the group size experienced by individuals (Jarman, 1974). High and significant values of this statistic, compared with those from random data sets, suggest the presence of individuals that are found in consistently larger or smaller groups than that of other individuals. Here, the HWI corrected by gregariousness, referred to as HWIG (Godde et al., 2013) was used. In the HWIG, the HWI between individuals *a* and *b* is divided by the sum of the HWIs involving *a* and the sum of those involving *b*, and multiplied by the sum of all association indices. This correction also changes the index interpretation because it is no longer restricted to between zero and one. A HWIG equals one means that a pair of individuals associate at random; a HWIG lower than one indicates that a pair associate less often than expected, and a HWIG higher than one indicates that a pair associate more often than expected, given their gregariousness (Godde et al., 2013).

Monte Carlo simulations were performed following the methodology proposed by Bejder et al. (1998) and modified by Whitehead et al. (2005), to verify if the associations between individuals of this population occur more frequently than expected by chance, and to find potential significant levels of association (preferred/avoided) between pairs of individuals. The sampling periods were defined as sampling occasions, which corresponded to one day, to avoid the influence of demographic effects during the study period (i.e., births, deaths, immigration, and emigration) (Whitehead & Dufault, 1999). The original matrix of association was randomized until the *p* value stabilized (in our case at 40,000 iterations), with 1,000 flips per permutation. This test suggests long‐term preferred companionships when the standard deviation (*SD*) of the real association indices are significantly higher than those expected by chance, whereas if mean of the real association indices is significantly lower than the random mean, this indicates short‐term preferred companionships (Whitehead, 2009). To verify if the collected data were sufficient for a good description of the social structure of this population, the social differentiation (*S*) and the correlation coefficient between the true association indices and their estimated values (*r*) were calculated using the methods described by Whitehead (2008b). The social differentiation indicates the variability of the association index within the population: if *S* is near 0, the relationships within the population are homogeneous; if *S* is close to or >1, the associations are highly variable and fewer associations are needed for detecting the preferred companionships (Whitehead, 2008b). The correlation coefficient between the true association indices and the calculated association indices (*r*) is a measure of precision of the representation to describe the social structure (the matrix of the association index) of a population, indicating how close it is to reality. Values of *r* near 1 indicate an excellent representation, whereas values close to 0 indicate a poor representation (Whitehead, 2008b). The standard errors were calculated through 10,000 bootstrap replications. All social and network structure analyses were run in SOCPROG, version 2.8 (Whitehead, 2009).

### 2.4 Constructing generalized affiliation indices (GAIS)

The GAIs were constructed using the half‐weight index (with gregariousness entered as one of the predictor measures) with a binomial model. The significance of the predictor variables was examined using the multiple regression quadratic assignment procedure (MRQAP). This test considers whether each of the predictor matrices, controlling for the presence of the other predictors, makes a significant contribution toward explaining the matrix of association indices. The MRQAP was performed with 20,000 permutations (using the “double‐semi‐partialing” technique of Dekker, Krackhardt, and Snijders 2007), and the effective contribution of each predictor was measured by the partial correlation coefficients. To identify particularly large positive or negative affiliations (greater/smaller than ±2.5; Whitehead and James 2015), the residuals of this procedure were transformed into Anscombe residuals (Pierce & Schafer, 1986). The calculated prediction measures were as follows:

#### 2.4.1 Gregariousness

Differently of the correction made in the HWI, gregariousness as a predictor variable was calculated following Whitehead and James's (2015) correction, where the gregariousness predictor between two individuals (*a* and *b*) is the log of the sum of the association indices involving *a* (except the *ab* index) multiplied by the sum of those involving *b* (except the *ba* index).

#### 2.4.2 Spatial and home range overlap

Individuals using the same area tend to associate more often with each other. To investigate spatial overlap, we calculated the proportion of those months in which both individuals in a pair were identified in the same area (estuary, northern coast, southern coast). Month was chosen as a period because of the survey procedure, which was intended to monitor all areas at least once every month. The home range overlap between pairs of individuals were estimated following the kernel‐based utilization distribution overlap index method (Fieberg & Kochanny, 2005), which is implemented in the package AdehabitatHR (Calenge, 2006) for R v 3.4.3 (R Core Team, 2013).

#### 2.4.3 Temporal overlap

Individuals using an area at the same time are more likely to be associated with each other. The study period corresponds to a total of ten years, which equates to 120 months. The temporal overlap was calculated as the sum of months that at least one individual of a pair was identified, divided by the sum of months that both were identified.

#### 2.4.4 Sex, area and period classes

Predictors were calculated for each class that was used in the Mantel tests with the HWIG. For that, it was constructed a *x*(attribute class)*_ij_* matrix for each class, where 1 is given if *i* and *j* have the same attribute and zero if they have a different attribute.

### 2.5 Detecting social units

The detection of social units was performed through modularity, which is the difference between the proportion of the total associations within clusters and the expected proportion, given the summed associations of the different individuals (Newman, 2004). In order to find the best delineation, Newman (2006) suggests an eigenvector‐based method as being generally efficient and this was implemented by SOCPROG and UCINET (Borgatti, Everett, & Freeman, 2002). This method is based on defining a parsimonious division of the individuals, which maximizes the weight and the number of associations within the units and consequently minimizes the associations between them. The modularity coefficient (*Q*) measures the quality of the division, observing if individuals are designated to clusters with many internal connections and few connections with other clusters, indicating a good division when *Q* is greater or equal to 0.3 (Newman & Girvan, 2004). The coefficient *Q* is the sum of all pairs of associations belonging to the same cluster, minus the expected value if the pairs were randomly associated, given the strength of the connection between the individuals. The spring embedding layout was used in NetDraw (Borgatti, 2002) to draw the social network diagram, showing only associations with HWIG > 1.

### 2.6 Network metrics

Network metrics are statistical measures used to characterize properties of an individual or a network as a whole (Farine & Whitehead, 2015). Three individual‐based network statistics, calculated from the weighted network (association matrix), were averaged over and within the social units: (a) strength, which is a measure of gregariousness, and is the sum of the association indices for each individual (Barthélemy, Barrat, Pastor‐Satorras, & Vespignani, 2005); (b) the clustering coefficient, which measures how well the partners of an individual are themselves associated (as calculated by Holme, Park, Kim, & Edling, 2007); and (c) affinity, which is higher when individuals are connected to other individuals with high strength (Whitehead, 2009). To verify whether the network structure was influenced by individual association preferences and/or whether association patterns differed significantly between social units, the calculated network metrics for each unit were compared to those of an expected network based on 10,000 permutations (Lusseau, Whitehead, & Gero, 2008).

### 2.7 Temporal patterns of association

Association indices represent the proportion of time that pairs of individuals were associated, but it does not distinguish whether and when associations were interrupted over a certain period of time. Thus, to assess temporal stability of associations, we calculated the standardized lagged association rate (SLAR) within the disclosed social units using the HWIG. SLAR is the estimated probability that a previously associated pair will be found in association after a given time lag, accounting for the fact that not all individuals within the groups were identified (Whitehead, 1995). We estimated the standard error of SLAR using a Jackknife procedure with 1,000 replications omitting 10 sampling periods each time (Whitehead, 2008b). As a theoretical benchmark, we compared the empirical SLAR with the null expectation, that is, when individuals associate at random (called standardized null association rate: SNAR). Results were plotted in a log‐scale of the sampling periods to better visualize decays.

In addition, we fitted four exponential decay models to the observed SLAR to possibly identify patterns in the association decay over time. These models contain parameters that can be interpreted as follows: preferred companions, where pairs of individuals have a preference for associating, which is constant over time; casual acquaintances, where pairs associate for some time, disassociate, and may reassociate; both preferred companions and casual acquaintances present; and two levels of casual acquaintances, where, for example, a stability of a pair changes from a short time scale to a longer one (Whitehead, 2008a). The most parsimonious model was selected based on the lowest value of the quasiAkaike information criterion (QAIC; Whitehead, 2007), with additional support of QAIC weights and likelihood (Burnham & Anderson, 2002).

## 3 RESULTS

During the study period, a total of 2,014 dolphin groups were encountered across 339 sampling occasions. During these encounters, 85,254 dorsal fin photographs were obtained, of which 51,920 were of Q1 quality, resulting in the identification of 217 individual dolphins. The mean observed group size was similar between the two coastal areas and the transition area, but slightly smaller in the estuary (Table 1). After data treatment for social analysis, 318 sampling occasions were considered; 1,792 groups fulfilled our requirements for inclusion (control of group size and minimum percentage of dolphins photographed in each group), with 102 dolphins used for further analysis based on established criteria. Data on the area classification, period classification and sex of the individuals used for analyses are presented in Supporting information Appendix S1: Table S1 and, for each area class, in Figure 1b–f. The classification of area created was suitable, since there were no cases of individuals who preferred two of the areas other than the coastal areas. In relation to the sexing of individuals, it was possible to determine the sex of 80 individuals (48 females and 32 males; Supporting information Appendix S1: Table S1).

**Table 1:** Group characteristics of Lahille's bottlenose dolphins (*Tursiops truncatus gephyreus*) sighted in 339 boat surveys realized between January 2006 and December 2015 in three subareas (Estuary, South, and North) and a transition area, in the Patos Lagoon estuary and adjacent coastal waters in southern Brazil

Subarea	No. of groups	Mean group size (*SD*)	Minimum and maximum number of individuals	Group size mode
Estuary	515	4.63 ± 4.13	1–27	2
South	393	7.27 ± 5.92	1–44	4
North	487	6.79 ± 5.08	1–29	3
Transition area	619	5.79 ± 4.92	1–35	3
Total	2014	6.02 ± 5.09	1–44	3

### 3.1 Social analysis

The coefficient of variation of the true association index using the likelihood method was relatively high (*S* = 0.891 ± 0.015), indicating a socially well‐differentiated population in which the relationships among individuals of the population are not necessarily homogeneous. The correlation between the true association index and the estimated association index (*r* = 0.642 ± 0.020) indicated that the analysis using association data among individuals had relatively good power to represent the true social system of this dolphin population. The “*SD* of the typical group size” was higher than expected by chance (real = 0.89, random = 0.74, *p*‐value = 0.0018). Therefore, the initial network was constructed using the HWIG, to avoid bias from the gregariousness of individuals. The association index among all pairs of individuals had a mean of 1.08 (*SD *= 0.27), with a maximum value of 39.98 (mean* *= 9.97, *SD *= 9.94). The permutation tests using the HWIG indicated that there is no long‐term (between sampling period) preferred companionships (*SD*_real_ **= 2.01 < *SD*_random_ **= 2.34 and CV_real_ **= 1.92 < CV_random_ **= 2.17, *p* = 0.999), but the lower proportion of nonzero association indices (real* *= 0.644, random* *= 0.705, *p* < 0.0001), which was significant, suggested that some individuals avoid others. Regarding the spatial (estuary, southern coast, northern coast, and nonpreferred area), period (cold, warm, and residents) and sex classification, which were used as covariates, the Mantel tests of these classes indicated that individuals with similar patterns of area use, period, and sex tended to associate more often with each other than with individuals with different patterns (*t* > 0 and *p* < 0.0001 for all three tests). This justifies the use of these classifications as predictors variables in the MRQAP.

### 3.2 Affiliation indices and predictors of social structure

**Figure 2:**
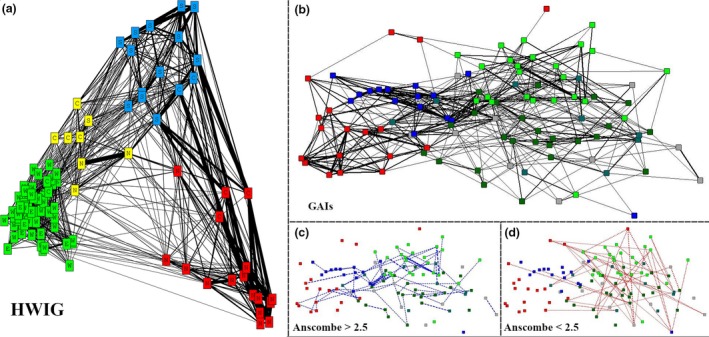
Network diagrams of 102 Lahille's bottlenose dolphins (*Tursiops truncatus gephyreus*) that use the Patos Lagoon Estuary and adjacent coastal waters in southern Brazil, using the half‐weight index corrected for gregariousness (a) and generalized affiliation indices (b). The thickness of the lines connecting each pair of individuals indicates the strength of their associations, and each node corresponds to an individual and their social unit (GR = social units proposed using HWIG; SU = social units proposed using GAIs; green variations = GR1/SUs1–4 individuals, yellow = GR4 individuals, blue = GR2/SU5 individuals, and red = GR3/SU6 individuals). Node labels correspond to the first letter of each spatial class: Wanderers, Estuary, South coast, North coast, and Coastal dolphins. High affiliations (Anscombe residuals > 2.5) and strong avoidance (Anscombe residuals < −2.5) were highlighted in (c) and (d), respectively.

Multiple regression quadratic assignment tests indicated that gregariousness, spatial overlap, and temporal overlap were useful predictors for explaining patterns of associations in this dolphin population (Table 2), but area class (significant *p*‐value (*p* = 0.0016), but with a low partial correlation), home range overlap, sex, and period were removed by the stepwise procedure. Therefore, GAIs were calculated using gregariousness, spatial overlap, and temporal overlap as predictor variables. The GAIs among all pairs of individuals had a mean 0.00 (*SD *= 0.01), with a maximum value of 0.55 (mean* *= 0.18, *SD *= 0.11). The permutation tests indicated that the mean association rate among all pairs of individuals (real* *= 0.00251, random* *= 0.00099, *p* < 0.0001) and the standard deviation (real* *= 0.038, random* *= 0.028, *p* < 0.0001) were significantly higher than expected, indicating the presence of long‐term preferred associations in the population. Large deviance residuals indicated 88 strongly affiliated associations, and low deviance residuals indicated 48 pairs with strong avoidance. Regarding the use of area classification, there were strong affiliations mostly within individuals of the same area class, and between southern and northern individuals (Figure 2c). Avoidances occurred mostly within wanderers, and between estuary and wanderer individuals (Figure 2d).

**Table 2:** Efficiency of predictor variables in explaining association indices between Lahille's bottlenose dolphins (*Tursiops truncatus gephyreus*), indicated by partial correlation coefficients and results of multiple regression quadratic assignment procedures (MQRAP) tests (10,000 replications)

Predictor	Partial correlation	MRQAP *p‐value*
Gregariousness	**−0.1722**	**0.0000**
Temporal overlap	**0.3383**	**0.0000**
Spatial overlap	**0.3457**	**0.0000**
Home range overlap	0.0098	0.7322
Area class	−0.0788	0.0016
Sex class	0.0255	0.1746
Period class	0.0089	0.7712

The used predictors are highlighted in bold

### 3.3 Detecting social units

Based on the HWIG, the estimated modularity coefficient (*Q*_max_ **= 0.364) suggests a reasonable division of the population into social units. The application of Newman's modularity (Newman, 2006) indicated four divisions in the population (Figure 2a), here called GRs units, and these were consistent with our area classification (Supporting information Appendix S1: Table S1). One unit was composed by at least 62 individuals that used the entire study area, though predominantly in the vicinities of the transition area (GR1). Two units were strongly associated with the coastal area; one in the southern coast (GR2) and one in the northern coast (GR3), with at least 15 and 17 dolphins, respectively. The uniqueness of these units is that most of the individuals do not use the inner estuary. The last unit is composed by at least 8 individuals that have preferences for the entire coastal area, but occasionally use the mouth of the estuary (GR4).

Removing spatiotemporal dynamics and gregariousness of the association index using GAIs, the estimated modularity coefficient was similar (*Q*_max_ **= 0.32), but instead of four, indicated six divisions (Figure 2b), here called social units (SUs). Although this index suggested a larger number of divisions in the population, the division mainly subdivided and reorganized individuals of the GR1 and GR4 units into four social units (SU1, SU2, SU3, and SU4). This implies that, in a scenario where spatiotemporal influence is excluded, individuals which composes the GR4 unit are no longer considered as important "connectors" between estuarine/wanderers and coastal individuals. The two social units associated with the coastal areas, SU5 and SU6, remained almost unchanged as the GR2 and GR3, respectively, with only three individuals designated to another social unit, and other three from other social units now designated as belonging to the coastal units. The SU6 maintained a clear separation from the other units and strong relationships among its individuals. On the other hand, the SU5, in the affiliation‐based diagram, seems to act as "connectors" between coastal and estuarine/wanderer dolphins. In terms of spatial and temporal patterns, the SUs 1, 2, 3, and 4 have almost the same home range and core areas, which correspond to the estuary mouth and coastal waters adjacent to the jetties (Figure 4a, b, c, and d, respectively), and are composed only by resident individuals. The SU5 and SU6 have distinct home ranges, with core areas adjacent to the transition area, but utilizing more the southern and northern coasts, respectively (Figure 4e, f). These units are composed by resident individuals that prefer the coastal areas and those transient individuals mostly found in the Cold or Warm periods. Regarding preferred affiliations in the social units, there were strong affiliations mostly within SU5 and SU6 (Figure 2c). Avoidances occurred mostly between SUs 1–4 individuals (Figure 2d).

### 3.4 Network metrics between social units

Using the HWIG and its putative units, both social units associated with the coastal area (GR2 and GR3) had similar and higher mean measures of strength, eigenvector centrality, clustering coefficient and affinity, than the overall means (Table 3). On the other hand, the GR1 and GR4, in general, presented lower mean measures than the overall means. Strength and eigenvector centrality measures using GAIs and their proposed units presented very similar results (Table 3). Unfortunately, the clustering coefficient and affinity measures using GAIs presented unreasonable standard errors, diminishing their interpretation. The lower mean strength and high eigenvector centrality in SU6 individuals, compared with the association‐based unit (GR3), reflect what is shown in the network diagrams (Figure 2). The strength within the SU6 is strong (mean* *= 0.94 ± 0.26), but its weaker relationships with the SUs1–4 individuals reduced its overall mean. This higher internal strength, in addition to the relationships with individuals of the SU5, which also have high strength values, explains the higher value of eigenvector centrality in the SU6. Differently to the SU6, the SU5 has more of a connector role inside the network and some individuals also associate with many individuals of the SUs1–4, which in turn have more fluid relationships. This likely explains the lower eigenvector centrality in the SU5.

**Table 3:** Mean strength, eigenvector centrality, clustering coefficient and affinity of individuals of each social unit, proposed using half‐weight index correct for gregariousness (HWIG; four GRs units) and generalized affiliation indices (GAIs; six SUs units), of the Lahille's bottlenose dolphin (*Tursiops truncatus gephyreus*) population that uses the Patos Lagoon Estuary and adjacent coastal waters in southern Brazil

Social Unit	Index	No. of ind.	Strength	Eigenvector centrality	Clustering coefficient	Affinity
GR1	HWIG	62	92.93 (3.39)	0.03 (0.01)	0.04 (0.001)	96.83 (1.92)
GR2	HWIG	15	127.26 (13.27)	0.11 (0.03)	0.10 (0.04)	121.68 (6.59)
GR3	HWIG	17	133.68 (12.11)	0.20 (0.06)	0.17 (0.09)	129.96 (8.93)
GR4	HWIG	8	106.26(9.75)	0.05 (0.01)	0.05 (0.01)	105.88 (3.76)
Overall means	HWIG	102	**105.82 (19.03)**	**0.07 (0.06)**	**0.07 (0.06)**	**106.72(14.35)**
SU1	GAIs	9	0.12 (0.10)	0.03 (0.01)	−0.60 (8.38)	−0.90 (5.23)
SU2	GAIs	10	−0.14 (0.06)	0.01 (0.04)	0.01 (1.26)	0.17 (3.64)
SU3	GAIs	24	0.18 (0.06)	0.05 (0.02)	−0.08 (7.50)	−1.33 (6.23)
SU4	GAIs	25	0.20 (0.08)	0.04 (0.02)	−0.29 (3.16)	−0.64 (7.39)
SU5	GAIs	16	0.81(0.42)	0.01 (0.03)	−0.12(1.84)	0.38(4.18)
SU6	GAIs	18	0.23(0.18)	0.17 (0.07)	−0.22(4.44)	0.96(2.62)
Overall means	GAIs	102	**0.25 (0.04)**	**0.06 (0.01)**	**−0.20 (2.12)**	**−0.30 (4.21)**

Correlation coefficients	HWIG 4 divisions	GAIs 6 divisions
Strength by clustering coefficient:	0.8268	0.0613
Strength by affinity:	0.9743	0.0291

Note

The standard deviation, estimated by bootstrap, is in brackets.

Overall means were highlighted in bold.

### 3.5 Temporal patterns of association

**Figure 3:**
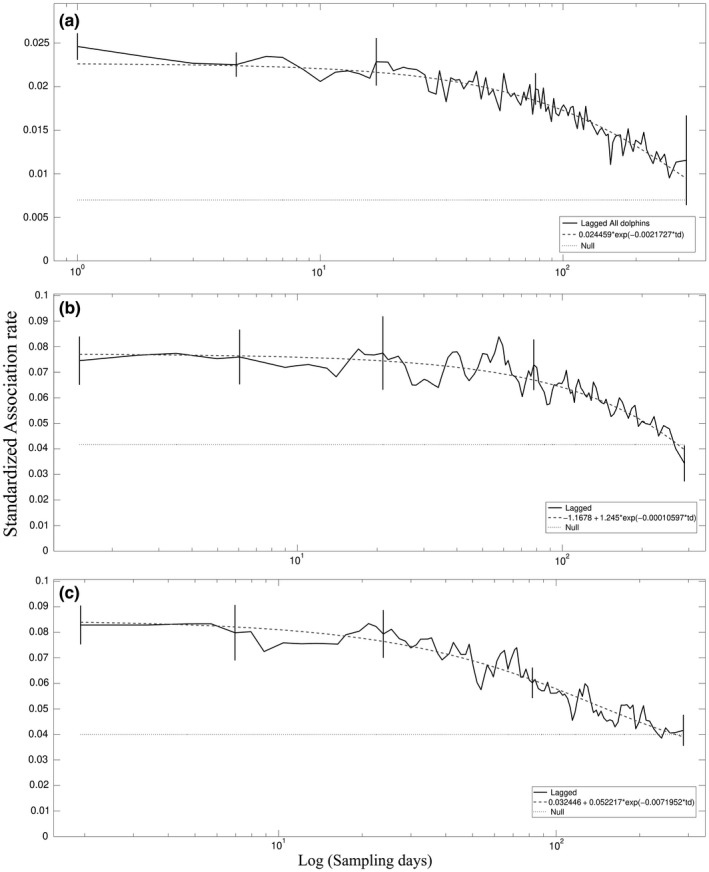
Standardized lagged association rate (solid line) compared to the best fitting model (dashed line) and standardized null association rate (dotted line) for all dolphins (a), within Social Unit 3 (b) and within Social Unit 4 (c) dolphins. Standard error bars (vertical lines) were computed by jackknifing and SLAR curves were smoothed with moving averages of 8,000 (a) and 5,000 (b, c) associations

The SLAR for all dolphins combined showed that the probability of recapture of individuals associated over time was low, decayed over time, but was still higher than expected by chance throughout the entire study period (Figure 3a). The error bars were relatively small, indicating the considerable precision of the estimates. The best fitting model consisted of casual acquaintances (Supporting information Appendix S1: Table S2). Despite the low probability of association between pairs, they still associated more often than expected by chance over more than 200 sampling periods (days) later. Considering the units suggested based on the GAIs separately, the SU3 and SU4 presented a similar pattern observed for the population (Figure 3b, c, respectively), differing due to the presence of preferred companions (Supporting information Appendix S1: Table S2). The probability of association between pairs is slightly higher (0.078), compared to the entire population (0.026), and the tendency of the pairs to dissociate is observed after 150 days (Figures 3b, c, respectively). The other social units (SUs, 2, 5, and 6) are composed of a smaller number of individuals, many of them with few sightings (compared with SUs3–4) and, therefore, their results are not presented.

**Figure 4:**
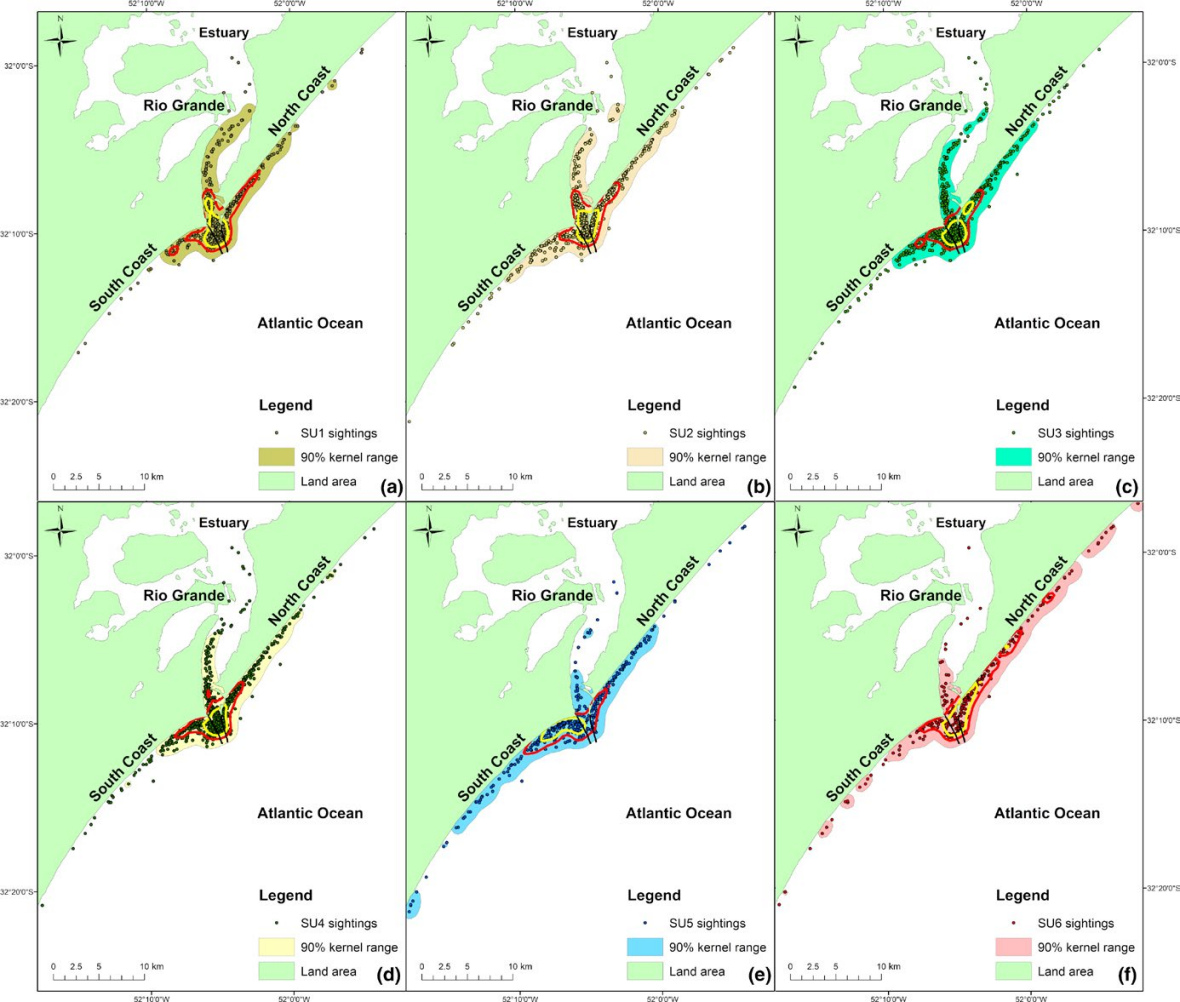
Locations of each social unit of Lahille's bottlenose dolphins (*Tursiops truncatus gephyreus*), proposed by community division and modularity based on generalized affiliation indices, with 90% (full color), 50% (red line), and 25% (yellow line) kernel isopleths. (a) Social Unit 1, (b) Social Unit 2, (c) Social Unit 3, (d) Social Unit 4, (e) Social Unit 5, and (f) Social Unit 6

## 4 DISCUSSION

Using ten years of photo‐ID data and social network analyses, this study showed that the Lahille's bottlenose dolphins inhabiting the Patos Lagoon estuary and adjacent coastal waters in southern Brazil show preferred and/or avoided associations and form social units likely driven by their gregariousness, spatiotemporal use patterns and social preferences. This pattern of social relationships and space/time use led to the identification of three major dolphin units or subpopulations based on spatial use patterns: a large unit composed by four affiliation‐based social units (SUs1–4) composed by resident individuals which use the entire study area but are mostly found in the estuary mouth and its adjacencies; and two coastal affiliation‐based social units (SU5 and SU6) composed by some residents, but with seasonal inputs from transient individuals, which, in general, do not use the inner estuary; one preferentially using the southern area, and the other the northern area. The detection of transient individuals, as well as the differentiated spatiotemporal use of individuals in this population made affiliations (GAIs) the most appropriate method to describe the social network of this population. Overall, this population presented a typical fission–fusion social dynamics, which was predominantly composed of pairs of casual acquaintances that maintained associations over a few days, as well as some long‐lasting associations and preferred companionships.

### 4.1 Ranging behavior

Spatial dynamics are important to consider when examining animal sociality, especially when studying animals which are capable of long‐range movements (10s–1,000s of km) in short periods of time (days–months) such as dolphins (Irvine, Scott, Wells, & Kaufmann, 1981; Mate et al., 1995). In our study, we identified social units composed by individuals that: (a) use the entire study area but mainly concentrate around the estuary mouth; (b) use mostly the inner estuary area but also use the coastal area; (c) use the entire coastal area; and (d) use mostly the coastal area north or south to the estuary mouth. This differentiated use of areas was reflected in the structure revealed by the association‐based (HWIG) network (Figure 2), which does not control for the effect of spatial overlap. This bias, by itself, justifies the use of GAIs to understand the true affiliations of this population. However, even with distinct spatial use, the core areas of the coastal units are very close to the estuary mouth, resulting in high spatial overlap between all units (Figure 4). Because of this high spatial overlap, we tested the frequency of occurrence of pairs of individuals in the same area as a predictor measure of “spatial overlap”, which proved to explain better the social network of this population than the home range overlap itself. The presence of social units that share large parts of their core areas reinforces the importance of the temporal overlap as a predictor variable.

There are some examples of bottlenose dolphin populations where, differently from this study, present social structuring with little or even no core area overlap between units (Louis et al., 2015; Titcomb, O'Corry‐Crowe, Hartel, & Mazzoil, 2015; Urian et al., 2009; Wiszniewski et al., 2009). However, a similar pattern of social units with high spatial overlap emerging due to social preferences in other dolphin populations can be seen, for example, in bottlenose dolphins in the east coast of Scotland (Lusseau et al., 2006), and Guiana dolphins in the eastern coast of Brazil (Cantor et al., 2012). The large part of the population which frequently uses the PLE, the SUs1–4, is very well studied in terms of their population parameters and has remained stable over the last decades (Castello & Pinedo, 1977; Dalla Rosa, 1999; Fruet et al., 2011; Fruet, Daura‐Jorge, et al., 2015a). The PLE is a protected, highly productive environment (Seeliger & Odebrecht, 2010), which provides favorable environmental conditions throughout the year for these dolphins, particularly for feeding and shelter (Fruet, Daura‐Jorge, et al., 2015a; Mattos et al., 2007; Secchi et al., 2016). The fact that the coastal dolphins were not observed to enter this area, with such favorable characteristics, is noteworthy. Intraspecific territoriality, which could explain this kind of beavior and is widely seen in other mammals (e.g., primates, Watts & Mitani, 2001; Williams, Pusey, Carlis, Farms, & Goddall, 2002; carnivores, Heinsohn, 1997; rodents, Gurnell, 1984), is absent in most marine mammal species and has been poorly reported in resident *Tursiops* populations (Pearson, 2011). For some unknown reason, it seems that most of the SUs1–4 and SU6 dolphins avoid using the same area (in the northern coast) at the same time. This became evident on two occasions where we observed that the approach of SU6 dolphins to areas nearby the estuary triggered porpoising of dolphins from SUs1–4 to the estuary area (R. C. Genoves and P. F. Fruet, personal observations).

### 4.2 Space and time matters

Combining the spatial behavior with the temporal measure, we revealed that spatiotemporal dynamics is a key structural variable in this social network. This is the major difference between the association‐based network, which is biased by spatiotemporal dynamics, and the affiliation‐based network structure observed, which exclude this source of bias. It is known that individuals using the same area associate more often (Shizuka et al., 2014) and individuals using the area at the same time are more likely to associate (Cantor et al., 2012). Therefore, the HWIG probably overestimated associations between pairs of individuals of the same GR unit, resulting in a clearer division in the association‐based compared to affiliation‐based network. In other words, if it were not for the use of GAIs, the social divisions present in dolphins that use the estuary (estuarine and wanderers) would not be detected. Regarding some factors that can potentially affect the temporal patterns, population growth and seasonal variability were identified as the major factors affecting the temporal variability in African and Asian elephant societies (Wittemyer et al., 2005; de Silva, Ranjeewa, & Kryazhimskiy, 2011, respectively). However, as previously mentioned, this dolphin population appears to have remained stable during the study period. Data treatment was controlled for death and the presence of newly marked individuals, and there were no observations of migration or emigration into the area. Furthermore, the number of transient individuals at each period was very similar, with 8 individuals in the “Cold period” and 11 in the “Warm period,” confirming that there was no evidence of demographic effect over the years or between periods.

The temporal analysis considering all individuals showed that associations were nonrandom and characterized by short‐term relationships (casual acquaintances), consistent with the presence of social units, which are segregated from each other to a certain degree. Furthermore, permutation and SLAR tests indicated the presence of some long‐term associations within the social units of the study population. In cetacean populations governed by fission–fusion dynamics, associations between individuals could range from short‐term associations with little or no structure (e.g., *Cephalorhynchus hectori*, Bräger, 1999; *Tursiops* spp., Vermeulen, 2018) to strong long‐term sex and/or age‐related alliances (e.g., *Tursiops* spp., Wells, 1991; Connor & Heithaus, 1999; Lusseau et al., 2003; *Hyperoodon ampullatus*, Gowans, Whitehead, & Hooker, 2001; *Grampus griseus*, Hartman, Visser, & Hendriks, 2008; *Globicephala macrorhynchus*, Mahaffy, Baird, Mcsweeney, Webster, & Schorr, 2015). This Lahille's bottlenose dolphin population appears to be between these two extremes, exhibiting a complex mix of social stability and change in both space and time. This dynamic is not exclusive to this population and is similar to its “neighbor” Lahille's bottlenose dolphin population, which also presents social units with high spatial overlap but, differently from this population, has a strong influence of social preferences due to feeding specialization (Daura‐Jorge et al., 2012). Furthermore, disregarding the comparatively lower spatial overlap between units, it is very similar in terms of habitat specialization, probability of association (0.026–0.022) and temporal pattern (casual acquaintances and constant companions) to the *T. truncatus* population of Normano‐Breton Gulf, France (Louis et al., 2015).

### 4.3 Social network

The connection between social units can occur through a few key individuals. These key individuals, known as brokers (sensu Lusseau & Newman, 2004), form relationships with individuals of different social units and thus can play a crucial role in maintaining the cohesion of the population's social network as a whole. They are important for transferring information at different levels of the population (Rendell & Whitehead, 2001), assisting with gene flow within, but can also potentially lead to the spread of diseases (Frère et al., 2010; Newman, 2002). Considering only the association‐based social network (Figure 2a), the GR4 individuals appeared to act as brokers in this population. However, the affiliation‐based social network suggests that the SU5 individuals are more important for connecting SU6 dolphins to the SUs1–4 dolphins (Figure 2b). SU5 presented several moderate affiliative relationships with individuals from the other units and showed stable and long‐lasting associations with some SU6 dolphins. The reason for this greater social proximity with the SU6 may be due to their greater use of the northern area during the warm period. This behavior increases the opportunities for these individuals to associate and may explain the decrease in the density of individuals that use the southern area during the warm period, as detected by Di Tullio et al (2015). The northern coastal unit showed stable and long‐lasting associations mostly between individuals of their own unit, demonstrating that this unit is more socially segregated than the others are to each other in the population.

The modular network configuration of this Lahille's bottlenose dolphin population, structured by social units, is comparable to other fission–fusion societies such as that of Asian elephants, *Elephas maximus* (de Silva et al., 2011), spotted hyenas, *Crocuta crocuta* (Holekamp, Smith, Strelioff, Horn, & Watts, 2012) and Galapagos sea lions, *Zalophus wollebaeki* (Wolf, Mawdsley, Trillmich, & James, 2007), where individuals tend to interact more with each other to cope with environment changes and social pressures. However, the presence of transient individuals in this population resembles the pattern observed in a population of Guiana dolphins from Brazil (Cantor et al., 2012), where social units were composed by long‐term resident individuals and others by transient individuals. Although the structure between this Guiana dolphin population and ours is generally similar, an important difference is that the transient Guiana dolphins occupied a peripheral position in their network and were more closely and strongly connected among themselves. In our population, the cold period individuals were strongly associated to the southern coast residents, composing the SU5, and the warm period individuals were strongly associated to the northern coast residents, composing the SU6. In addition, dolphins that use the entire area (SUs 1, 2, 3, and 4) are more closely associated to the southern dolphins (SU5) than to the northern coast dolphins (SU6). This scenario suggests that transient cold period dolphins (that include some individuals sighted in Uruguayan waters by Laporta et al. 2016), which associated with SU5 individuals, are more socially connected to SUs1–4 than warm period transient individuals, who are more socially connected to SU6 dolphins. While this pattern can be mainly driven by social preferences, this hypothesis needs to be further explored by longer‐term studies including additional sightings of transient individuals. This could be achieved over the next few years but may be enhanced by increasing the survey effort and size of the area sampled in the coastal zone. The lower deviance residuals identified several avoidance relationships, mostly between individuals that use the estuary waters (estuarine and wanderer dolphins). This helps to explain why, even using almost the same area, these individuals compose four social units (SUs1–4). On the other hand, preferred relationships seem to be particularly important for the maintenance of the SU5 and SU6. Network metrics corroborated this, since dolphins that preferentially use the coastal area tend to have stronger relationships among themselves compared to dolphins that use the estuary or the entire area. Dolphins that were observed to use the inner estuary, but also use the coastal area, and those which use the entire study area (without particular area preference) have a greater chance of meeting and associating with other dolphins compared to those that show space use preferences over a smaller area (in relation to the study area; e.g., SU5 and SU6); this could explain the lower values of strength estimated for the SUs1–4. Another important characteristic was the low clustering coefficient (<0.2) for the population as a whole, which was particularly low for the SUs1–4 (Table 3), but similar to the Lahille's neighbor bottlenose dolphin population of Laguna (Daura‐Jorge et al., 2012) and an Indo‐Pacific botlenose dolphin population of Port Stephens, eastern Australia (Wiszniewski et al., 2009). Clustering coefficients are lower in territorial societies where individuals only associate with their neighbors, who, in turn, may not associate with each other (Whitehead, 2008a), which relates to the segregation by area observed in our study.

Our study on this Lahile's bottlenose dolphin population provides a better understanding of the impact of spatiotemporal dynamics and gregariousness on the patterns of social connections, but there are other structural variables that can also affect the social network. Genetic relatedness between individuals, for example, is a factor that is known to affect associations between individuals in many mammalian societies (e.g., spotted hyaenas, Wahaj et al., 2004; African elephants, *Loxodonta africana*, Archie, Moss, & Alberts, 2006; and Indo‐Pacific bottlenose dolphins, Wiszniewski et al., 2010), and should therefore be investigated. While we did not observe distinct feeding techniques in this population, the three subareas of the study show different ecological and physicochemical characteristics so it is possible that there are differences in the feeding ecology of the social units identified here (as observed for bottlenose dolphins of Normano‐Breton Gulf; Louis et al., 2018).

## 5 CONCLUSION

The Lahille's bottlenose dolphin population of the Patos Lagoon estuary and adjacent coast in Southern Brazil has a society which combines the fluid associations of a fission–fusion system with the affiliative structure of six social units and these appear to be mainly driven by social and spatiotemporal patterns. Our results demonstrate that even with high home range overlap, including core areas, individuals can use the same area at different times. This, added to the presence of transient individuals in different seasons (cold and warm), led the generalized affiliations indices to be the best choice to describe this complex social network. Preferred relationships between individuals had an important impact on the social network, increasing the cohesion of individuals in each social unit, particularly in the coastal units. Avoided relationships occurred mostly between resident dolphins, impacting on their subdivision. Transient individuals mostly associated with coastal residents when they were using the same area. Until other structural variables are not tested, the compilation of these results suggests that the social network of this population is mainly governed by social relationships impacted by spatiotemporal use patterns. Future studies including structural variables such as genetic relatedness and “feeding ecology” will contribute toward a better understanding of the drivers of this social structure. We recommend that the social units identified here should be used as a framework for modeling the dynamics and viability of this population, as well as for investigating patterns of gene flow within and between social units.

## ETHICAL STATEMENT

All applicable international, national, and/or institutional guidelines for the care and use of animals were followed. All procedures performed in studies involving animals were in accordance with the ethical standards of the institution or practice at which the studies were conducted. We only sampled adult animals and biopsy sampling procedures followed international guidelines, in accordance with ethical standards and under regional permits (Brazil's SISBIO 24,407–2, issued to P.F.F.). This article does not contain any studies with human participants performed by any of the authors.

## CONFLICT OF INTEREST

The authors declare that they have no conflict of interest.

## AUTHOR CONTRIBUTIONS

Rodrigo C. Genoves contributed substantially to the conception, data collection, analysis, interpretation, and drafting the work; Pedro F. Fruet and Juliana C. Di Tullio contributed substantially to the conception, data collection, drafting, and revising it critically for important intellectual content; Eduardo R. Secchi and Luciana Möller contributed substantially to the conception, drafting, and revising it critically for important intellectual content.

## DATA ACCESSIBILITY

Analysis reported in this article can be reproduced using the data provided in the Dryad Digital Repository: https://doi.org/10.5061/dryad.r8f277f.
