# Supplementary material for: Spatiotemporal use predicts social partitioning of bottlenose dolphins with strong home range overlap
Source: Ecol Evol. 2018 Dec 11;8(24):12597–614. doi: 10.1002/ece3.4681 (PMC6309009; doi:10.1002/ece3.4681)
Supplement: Supplementary file 1 [file ECE3-8-12597-s001.doc]

Appendix S1: Table S1. Characterization of the 102 Lahille’s bottlenose dolphins (*Tursiops truncatus gephyreus*), sorted and grouped based on the affiliation-based social units (CMD GAIs column), that fulfilled the prerequisites and were used in the social analyses. Abbreviation corresponds to: Females (F), Males (M) and Unknown (U) sex; DNA = Genetic method, PAC = Parental care method, DOF = dorsal fin method; Wanderer (WA), Estuary (ES), Southern (ES), Northern (NO) and Coastal (CO) dolphin; Cold = percentage of sightings in cold periods and Warm = percentage of sightings in warm periods; Resident (RES), Cold period (COL), Warm period (WAR), and cold period dolphin that was sighted in Uruguayan waters (COU); CMD-HWIG and GAIs = community division by modularity using the half-weight index corrected for gregariousness and generalized association indices (GAIs), respectively.

| ID | Sex | Sexing  Method | N. sampling  periods | Estuary  (%) | Southern  Coast (%) | Northern  Coast (%) | Area  Class | N.  Seasons | Cold  (%) | Warm  (%) | Period  Class | CMD  HWIG | CMD  GAIs |
| --- | --- | --- | --- | --- | --- | --- | --- | --- | --- | --- | --- | --- | --- |
| ID004 | F | DNA | 39 | 56.4 | 25.6 | 17.9 | ES | 18 | 44 | 56 | RES | GR1 | SU1 |
| ID013 | F | DNA | 63 | 34.9 | 30.2 | 34.9 | WA | 19 | 53 | 47 | RES | GR1 | SU1 |
| ID029 | M | DNA | 100 | 37.0 | 31.0 | 32.0 | WA | 20 | 50 | 50 | RES | GR1 | SU1 |
| ID033 | F | DNA | 80 | 38.8 | 21.3 | 40.0 | WA | 20 | 50 | 50 | RES | GR1 | SU1 |
| ID043 | F | PAC | 18 | 5.6 | 22.2 | 72.2 | NO | 14 | 50 | 50 | RES | GR4 | SU1 |
| ID078 | F | DNA | 13 | 0.0 | 27.3 | 72.7 | NO | 5 | 60 | 40 | RES | GR4 | SU1 |
| ID125 | U | --- | 31 | 51.6 | 25.8 | 22.6 | ES | 8 | 50 | 50 | RES | GR1 | SU1 |
| ID300 | F | PAC | 62 | 53.2 | 21.0 | 25.8 | ES | 18 | 44 | 56 | RES | GR1 | SU1 |
| ID301 | F | PAC | 73 | 60.3 | 13.7 | 26.0 | ES | 19 | 47 | 53 | RES | GR1 | SU1 |
| ID001 | F | DNA | 121 | 62.8 | 11.6 | 25.6 | ES | 20 | 50 | 50 | RES | GR1 | SU2 |
| ID002 | F | PAC | 88 | 65.9 | 10.2 | 23.9 | ES | 19 | 53 | 47 | RES | GR1 | SU2 |
| ID008 | M | DNA | 78 | 34.6 | 35.9 | 29.5 | WA | 18 | 44 | 56 | RES | GR1 | SU2 |
| ID025 | F | DNA | 90 | 50.0 | 23.3 | 26.7 | ES | 20 | 50 | 50 | RES | GR1 | SU2 |
| ID056 | F | PAC | 59 | 37.3 | 18.6 | 44.1 | WA | 20 | 50 | 50 | RES | GR1 | SU2 |
| ID063 | M | DNA | 48 | 0.0 | 0.0 | 100.0 | NO | 17 | 47 | 53 | RES | GR3 | SU2 |
| ID090 | M | DNA | 47 | 0.0 | 0.0 | 100.0 | NO | 15 | 47 | 53 | RES | GR3 | SU2 |
| ID092 | U | --- | 62 | 1.6 | 83.9 | 14.5 | SO | 14 | 43 | 57 | RES | GR2 | SU2 |
| ID120 | M | DNA | 94 | 46.8 | 13.8 | 39.4 | WA | 14 | 50 | 50 | RES | GR1 | SU2 |
| ID196 | M | DNA | 33 | 15.2 | 36.4 | 48.5 | WA | 4 | 50 | 50 | RES | GR1 | SU2 |
| ID006 | F | DNA | 69 | 75.4 | 1.4 | 23.2 | ES | 13 | 46 | 54 | RES | GR1 | SU3 |
| ID007 | F | PAC | 62 | 27.4 | 43.5 | 29.0 | WA | 19 | 53 | 47 | RES | GR1 | SU3 |
| ID012 | F | DNA | 75 | 24.0 | 33.3 | 42.7 | WA | 20 | 50 | 50 | RES | GR1 | SU3 |
| ID014 | F | PAC | 65 | 56.9 | 14.1 | 29.0 | ES | 20 | 50 | 50 | RES | GR1 | SU3 |
| ID016 | M | DNA | 48 | 22.9 | 39.6 | 37.5 | WA | 15 | 47 | 53 | RES | GR1 | SU3 |
| ID019 | F | DNA | 66 | 25.8 | 25.8 | 48.5 | WA | 18 | 56 | 44 | RES | GR1 | SU3 |
| ID023 | M | DNA | 64 | 15.6 | 50.0 | 34.4 | WA | 18 | 50 | 50 | RES | GR1 | SU3 |
| ID024 | F | DNA | 41 | 34.1 | 31.7 | 34.1 | WA | 13 | 46 | 54 | RES | GR1 | SU3 |
| ID037 | F | DNA | 47 | 0.0 | 40.4 | 59.6 | CO | 18 | 44 | 56 | RES | GR4 | SU3 |
| ID038 | M | DNA | 58 | 5.2 | 39.7 | 55.2 | WA | 17 | 41 | 59 | RES | GR1 | SU3 |
| ID039 | F | DNA | 76 | 43.4 | 19.7 | 36.8 | WA | 19 | 47 | 53 | RES | GR1 | SU3 |
| ID047 | F | DNA | 78 | 25.6 | 38.5 | 35.9 | WA | 20 | 50 | 50 | RES | GR1 | SU3 |
| ID059 | F | DNA | 89 | 42.7 | 14.6 | 42.7 | WA | 20 | 50 | 50 | RES | GR1 | SU3 |
| ID066 | M | DNA | 72 | 0.0 | 44.4 | 55.6 | CO | 20 | 50 | 50 | RES | GR1 | SU3 |
| ID079 | U | --- | 64 | 28.1 | 26.6 | 45.3 | WA | 18 | 50 | 50 | RES | GR1 | SU3 |
| ID105 | M | DNA | 74 | 24.3 | 31.1 | 44.6 | WA | 19 | 47 | 53 | RES | GR1 | SU3 |
| ID107 | F | DNA | 97 | 47.4 | 20.6 | 32.0 | WA | 18 | 50 | 50 | RES | GR1 | SU3 |
| ID118 | U | --- | 82 | 57.3 | 14.6 | 28.0 | ES | 19 | 53 | 47 | RES | GR1 | SU3 |
| ID127 | F | DNA | 68 | 0.0 | 39.7 | 60.3 | CO | 14 | 50 | 50 | RES | GR4 | SU3 |
| ID131 | U | --- | 40 | 20.0 | 42.5 | 37.5 | WA | 9 | 44 | 56 | RES | GR1 | SU3 |
| ID142 | F | DNA | 63 | 15.9 | 42.9 | 41.3 | WA | 17 | 53 | 47 | RES | GR1 | SU3 |
| ID172 | M | DNA | 47 | 8.5 | 40.4 | 51.1 | WA | 17 | 47 | 53 | RES | GR1 | SU3 |
| ID177 | M | DNA | 46 | 0.0 | 54.3 | 45.7 | CO | 15 | 53 | 47 | RES | GR1 | SU3 |
| ID198 | U | --- | 55 | 30.9 | 29.1 | 40.0 | WA | 15 | 53 | 47 | RES | GR1 | SU3 |
| ID005 | M | DNA | 72 | 40.3 | 27.8 | 31.9 | WA | 19 | 47 | 53 | RES | GR1 | SU4 |
| ID010 | F | DNA | 54 | 46.3 | 16.7 | 37.0 | WA | 18 | 56 | 44 | RES | GR1 | SU4 |
| ID011 | F | PAC | 69 | 23.2 | 21.7 | 55.1 | WA | 20 | 50 | 50 | RES | GR1 | SU4 |
| ID015 | F | DNA | 64 | 65.6 | 14.1 | 20.3 | ES | 20 | 50 | 50 | RES | GR1 | SU4 |
| ID017 | M | DNA | 51 | 19.6 | 31.4 | 49.0 | WA | 18 | 50 | 50 | RES | GR1 | SU4 |
| ID020 | M | DNA | 71 | 29.6 | 42.3 | 28.2 | WA | 20 | 50 | 50 | RES | GR1 | SU4 |
| ID022 | F | DNA | 65 | 18.5 | 40.0 | 41.5 | WA | 19 | 53 | 47 | RES | GR1 | SU4 |
| ID027 | M | DNA | 58 | 34.5 | 29.3 | 36.2 | WA | 19 | 47 | 53 | RES | GR1 | SU4 |
| ID031 | F | DNA | 46 | 17.4 | 45.7 | 37.0 | WA | 16 | 44 | 56 | RES | GR1 | SU4 |
| ID034 | M | DNA | 58 | 31.0 | 34.5 | 34.5 | WA | 18 | 44 | 56 | RES | GR1 | SU4 |
| ID041 | F | PAC | 101 | 36.6 | 31.7 | 31.7 | WA | 20 | 50 | 50 | RES | GR1 | SU4 |
| ID045 | F | DNA | 66 | 28.8 | 28.8 | 42.4 | WA | 19 | 47 | 53 | RES | GR1 | SU4 |
| ID048 | F | DNA | 76 | 32.9 | 39.5 | 27.6 | WA | 19 | 47 | 53 | RES | GR1 | SU4 |
| ID055 | F | DNA | 65 | 35.4 | 24.6 | 40.0 | WA | 20 | 50 | 50 | RES | GR1 | SU4 |
| ID058 | F | PAC | 38 | 5.3 | 28.9 | 65.8 | WA | 18 | 56 | 44 | RES | GR1 | SU4 |
| ID071 | F | PAC | 46 | 0.0 | 69.6 | 30.4 | CO | 16 | 56 | 44 | RES | GR4 | SU4 |
| ID091 | F | PAC | 118 | 63.6 | 19.5 | 16.9 | ES | 20 | 50 | 50 | RES | GR1 | SU4 |
| ID104 | F | PAC | 63 | 9.5 | 49.2 | 41.3 | WA | 17 | 47 | 53 | RES | GR1 | SU4 |
| ID115 | U | --- | 41 | 0.0 | 61.0 | 39.0 | CO | 17 | 53 | 47 | RES | GR4 | SU4 |
| ID116 | F | DNA | 46 | 39.1 | 15.2 | 45.7 | WA | 20 | 50 | 50 | RES | GR1 | SU4 |
| ID176 | M | DNA | 58 | 29.3 | 32.8 | 37.9 | WA | 15 | 47 | 53 | RES | GR1 | SU4 |
| ID179 | M | DNA | 45 | 62.2 | 20.0 | 17.8 | ES | 8 | 50 | 50 | RES | GR1 | SU4 |
| ID197 | F | DNA | 30 | 23.3 | 36.7 | 40.0 | WA | 8 | 50 | 50 | RES | GR1 | SU4 |
| ID214 | F | DNA | 74 | 21.6 | 35.1 | 43.2 | WA | 17 | 53 | 47 | RES | GR1 | SU4 |
| ID302 | F | PAC | 31 | 3.2 | 9.7 | 87.1 | NO | 17 | 53 | 47 | RES | GR4 | SU4 |
| ID030 | M | DNA | 95 | 38.9 | 27.4 | 33.7 | WA | 20 | 50 | 50 | RES | GR1 | SU5 |
| ID054 | M | DOF | 47 | 19.1 | 61.7 | 19.1 | SO | 18 | 44 | 56 | RES | GR4 | SU5 |
| ID062 | U | --- | 19 | 0.0 | 47.4 | 52.6 | CO | 13 | 54 | 46 | RES | GR2 | SU5 |
| ID067 | F | DNA | 19 | 0.0 | 84.2 | 15.8 | SO | 10 | 90 | 10 | COU | GR2 | SU5 |
| ID070 | M | DNA | 41 | 0.0 | 70.7 | 29.3 | SO | 11 | 45 | 55 | RES | GR2 | SU5 |
| ID072 | U | --- | 28 | 0.0 | 100.0 | 0.0 | SO | 10 | 70 | 30 | COU | GR2 | SU5 |
| ID074 | U | --- | 29 | 0.0 | 100.0 | 0.0 | SO | 8 | 75 | 25 | COL | GR2 | SU5 |
| ID084 | U | --- | 20 | 0.0 | 100.0 | 0.0 | SO | 8 | 88 | 13 | COL | GR2 | SU5 |
| ID085 | U | --- | 30 | 0.0 | 70.0 | 30.0 | SO | 14 | 57 | 43 | RES | GR2 | SU5 |
| ID089 | M | DNA | 29 | 0.0 | 100.0 | 0.0 | SO | 11 | 73 | 27 | COU | GR2 | SU5 |
| ID093 | M | DOF | 34 | 0.0 | 61.8 | 38.2 | CO | 14 | 29 | 71 | WAR | GR2 | SU5 |
| ID103 | U | --- | 44 | 0.0 | 70.5 | 29.5 | SO | 12 | 50 | 50 | RES | GR2 | SU5 |
| ID113 | U | --- | 12 | 0.0 | 91.7 | 8.3 | SO | 7 | 71 | 29 | COU | GR2 | SU5 |
| ID141 | M | DNA | 10 | 0.0 | 100.0 | 0.0 | SO | 7 | 71 | 29 | COU | GR2 | SU5 |
| ID150 | U | --- | 11 | 0.0 | 100.0 | 0.0 | SO | 4 | 75 | 25 | COL | GR2 | SU5 |
| ID174 | U | --- | 33 | 0.0 | 24.2 | 75.8 | NO | 9 | 44 | 56 | RES | GR3 | SU5 |
| ID049 | F | PAC | 77 | 31.2 | 20.8 | 48.1 | WA | 20 | 50 | 50 | RES | GR1 | SU6 |
| ID069 | M | DNA | 71 | 32.4 | 35.2 | 32.4 | WA | 19 | 47 | 53 | RES | GR1 | SU6 |
| ID094 | F | DNA | 15 | 0.0 | 0.0 | 100.0 | NO | 12 | 42 | 58 | RES | GR3 | SU6 |
| ID095 | F | PAC | 44 | 0.0 | 0.0 | 100.0 | NO | 16 | 44 | 56 | RES | GR3 | SU6 |
| ID096 | F | DNA | 33 | 0.0 | 39.4 | 60.6 | CO | 15 | 47 | 53 | RES | GR3 | SU6 |
| ID097 | F | DNA | 36 | 0.0 | 0.0 | 100.0 | NO | 14 | 29 | 71 | WAR | GR3 | SU6 |
| ID123 | M | DNA | 58 | 25.9 | 24.1 | 50.0 | WA | 13 | 46 | 54 | RES | GR1 | SU6 |
| ID143 | M | DNA | 11 | 0.0 | 36.4 | 63.6 | CO | 4 | 25 | 75 | WAR | GR3 | SU6 |
| ID145 | M | DNA | 10 | 0.0 | 0.0 | 100.0 | NO | 5 | 20 | 80 | WAR | GR3 | SU6 |
| ID147 | U | --- | 10 | 0.0 | 0.0 | 100.0 | NO | 5 | 20 | 80 | WAR | GR3 | SU6 |
| ID159 | F | DNA | 14 | 0.0 | 92.9 | 7.1 | SO | 4 | 50 | 50 | RES | GR2 | SU6 |
| ID160 | M | DNA | 10 | 0.0 | 0.0 | 100.0 | NO | 4 | 25 | 75 | WAR | GR3 | SU6 |
| ID166 | U | --- | 13 | 0.0 | 53.8 | 46.2 | CO | 7 | 29 | 71 | WAR | GR3 | SU6 |
| ID167 | U | --- | 10 | 0.0 | 0.0 | 100.0 | NO | 4 | 25 | 75 | WAR | GR3 | SU6 |
| ID169 | M | DNA | 10 | 0.0 | 0.0 | 100.0 | NO | 4 | 25 | 75 | WAR | GR3 | SU6 |
| ID170 | U | --- | 15 | 0.0 | 13.3 | 86.7 | NO | 7 | 29 | 71 | WAR | GR3 | SU6 |
| ID171 | U | --- | 13 | 0.0 | 0.0 | 100.0 | NO | 7 | 29 | 71 | WAR | GR3 | SU6 |
| ID184 | U | --- | 10 | 0.0 | 16.7 | 83.3 | NO | 4 | 25 | 75 | WAR | GR3 | SU6 |
