# Supplementary material for: Spatiotemporal use predicts social partitioning of bottlenose dolphins with strong home range overlap
Source: Ecol Evol. 2018 Dec 11;8(24):12597–614. doi: 10.1002/ece3.4681 (PMC6309009; doi:10.1002/ece3.4681)
Supplement: Supplementary file 2 [file ECE3-8-12597-s002.doc]

Appendix S1: Table S2. Models fit to Standardized Lagged Association Rates (SLAR) ranked by the lowest quasi-Akaike Information Criteria (QAIC) for all Lahille’s bottlenose dolphins (*Tursiops truncatus gephyreus*) and for each of the proposed social units (SUs) using generalized affiliation indices, of the Patos Lagoon Estuary and adjacent coastal waters in southern Brazil. Preferred companions (Pref. Comps), casual acquaintances (Casual acqs), both preferred companions and casual acquaintances present (Pref + casual) and two levels of casual acquaintances (Two levels) were the four models fitted. ∆QAIC, QAIC weights and Likelihood indicates the relative support for each model.

| **SLAR model** | **Model formula** | | **QAIC** | | **∆QAIC** | | **QAIC weight** | | **Likelihood** | |  |
| --- | --- | --- | --- | --- | --- | --- | --- | --- | --- | --- | --- |
| **All individuals** |  | |  | |  | |  | |  | |  |
| **Casual acqs** | 0.024**e*^(–0.0022∙t)^ | | 405,410.13 | | 0 | | 0.88 | | 1 | |  |
| **Two levels** | -0.63**e*^(–0.0022∙t)^+0.66**e*^(–0.0022∙t)^ | | 405,414.13 | | 4.0 | | 0.12 | | 0.13 | |  |
| **Pref + casual** | 0.019+0.029**e*^(–1.25∙t)^ | | 406,350.36 | | 940.2 | | 0 | | 0 | |  |
| **Pref. Comps** | 0.019974 | | 406,403.52 | | 993.4 | | 0 | | 0 | |  |
| **SU1 x SU1** |  | |  | |  | |  | |  | | |
| **Two levels** | 0.343**e*^(-0.007*t)^+0.02**e*^(-0.007*t)^ | | 11,911.59 | | 0 | | 0.99 | | 1 | |  |
| **Pref + casual** | 0.184+0.183**e*^(–0.017*t)^ | | 11,930.35 | | 18.75 | | 0 | | 0 | |  |
| **Casual acqs** | 0.308**e*^(-0.002*t)^ | | 11,977.67 | | 66.07 | | 0 | | 0 | |  |
| **Pref. Comps** | 0.242 | | 12,091.22 | | 179 | | 0 | | 0 | |  |
| **SU2 x SU2** |  | |  | |  | |  | |  | | |
| **Two levels** | -1.142**e*^(-0.013*t)^+1.47**e*^(-0.009*t)^ | | 21,321.98 | | 0 | | 0.99 | | 1 | |  |
| **Pref + casual** | -6.79+7.18**e*^(–0.0001*t)^ | | 21,351.33 | | 29.35 | | 0 | | 0 | |  |
| **Casual acqs** | 0.403**e*^(-0.004*t)^ | | 21,382.30 | | 60.31 | | 0 | | 0 | |  |
| **Pref. Comps** | 0.286 | | 21,899.47 | | 577 | | 0 | | 0 | |  |
| **SU3 x SU3** |  | |  | |  | |  | |  | | |
| **Pref + casual** | -1.167+1.24**e*^(–0.0001*t)^ | | 11,3542.20 | | 0 | | 0.93 | | 1 | |  |
| **Casual acqs** | 0.078**e*^(-0.002*t)^ | | 11,3547.74 | | 5.53 | | 0.06 | | 0.006 | |  |
| **Two levels** | -0.0185**e*^(-1.9*t)^+0.078**e*^(-0.002*t)^ | | 11,3551.51 | | 9.3 | | 0.008 | | 0.009 | |  |
| **Pref. Comps** | 0.0651 | | 11,3856.85 | | 314 | | 0 | | 0 | |  |
| **SU4 x SU4** |  | |  | |  | |  | |  | | |
| **Pref + casual** | 0.032+0.05**e*^(–0.007*t)^ | | 71,098.35 | | 0 | | 0.98 | | 1 | |  |
| **Casual acqs** | 0.08**e*^(-0.003*t)^ | | 71,106.72 | | 8.36 | | 0.01 | | 0.01 | |  |
| **Two levels** | 2.287**e*^(-6*t)^+0.08**e*^(-0.003*t)^ | | 71,110.16 | | 11.8 | | 0.002 | | 0.002 | |  |
| **Pref. Comps** | 0.0617 | | 71,536.00 | | 437 | | 0 | | 0 | |  |
| **SU5 x SU5** | |  | |  | |  | |  | |  | |
| **Casual acqs** | | 0.095**e*^(-0.005*t)^ | | 3,059.99 | | 0 | | 0.77 | | 1 | |
| **Two levels** | | 0.04**e*^(-0.99*t)^+0.091**e*^(-0.004*t)^ | | 3,063.27 | | 3.3 | | 0.15 | | 0.19 | |
| **Pref. Comps** | | 0.079 | | 3,065.97 | | 6.0 | | 0.038 | | 0.05 | |
| **Pref + casual** | | 0.077+0.058**e*^(–0.62*t)^ | | 3,066.14 | | 6.2 | | 0.035 | | 0.04 | |
| **SU6 x SU6** | |  | |  | |  | |  | |  | |
| **Pref + casual** | | 0.13-0.206**e*^(–0.045*t)^ | | 2,756.23 | | 0 | | 0.91 | | 1 | |
| **Casual acqs** | | 0.29**e*^(-0.011*t)^ | | 2,761,51 | | 5.3 | | 0.064 | | 0.07 | |
| **Two levels** | | 0.241**e*^(-0.004*t)^+0.28**e*^(-0.011*t)^ | | 2,763.83 | | 7.6 | | 0.02 | | 0.02 | |
| **Pref. Comps** | | 0.207 | | 2,807.10 | | 50.9 | | 0 | | 0 | |
|  |  | |  | |  | |  | |  | |  |
